# Effect of erbium, chromium-doped: yttrium, scandium, gallium, and garnet laser-assisted periodontal therapy using radial firing tip during early healing period: a randomized controlled split-mouth clinical trial

**DOI:** 10.1186/s12903-024-04270-1

**Published:** 2024-05-08

**Authors:** Jung Soo Park, Hannah Jung, Jae-Jun Ryu, Ki-Tae Koo, Jaebum Lee

**Affiliations:** 1 Department of Periodontology, Korea University Anam Hospital, Seoul, Republic of Korea; 2 Department of Biostatistics, Korea University College of Medicine, Seoul, Republic of Korea; 3 Department of Prosthodontics, Korea University Anam Hospital, Seoul, Republic of Korea; 4 School of Dentistry and Dental Research Institute, Seoul National University, Seoul, Republic of Korea; 5 Laboratory for Applied Periodontal & Craniofacial Research, Adams School of Dentistry, University of North Carolina, Chapel Hill, USA

**Keywords:** Laser, Periodontal treatment

## Abstract

**Background:**

This study aimed to demonstrate the efficacy of erbium, chromium-doped:yttrium, scandium, gallium, and garnet (Er,Cr:YSGG) laser-assisted nonsurgical periodontal therapy in periodontitis patients during 8 weeks of healing.

**Methods:**

A split-mouth, single-blinded, randomized controlled clinical trial was conducted on 12 patients diagnosed with stage III/IV periodontitis and had a minimum of two teeth with probing pocket depth (PPD) > 5 mm in at least two quadrants. Upon randomization, each quadrant was assigned for conventional scaling and root planing (SRP) procedure or laser-assisted therapy (SRP + laser) using radial firing tip (RFPT 5, Biolase). Clinical measurements and gingival crevicular fluid collection were performed for statistical analysis.

**Results:**

In the initial statistical analysis on the whole subject teeth, modified gingival index (MGI) reduction was greater in test group at 1(*P* = 0.0153), 4 (*P* = 0.0318), and 8 weeks (*P* = 0.0047) compared to the control in the same period. PPD reduction at 4 weeks in test group was -1.67 ± 0.59 showing significant difference compared to the control (-1.37 ± 0.63, *P* = 0.0253). When teeth with mean PPD ≥5 mm were sorted, MGI decrease was significantly greater in test group at 1 (*P*=0.003) and 8 week (*P*=0.0102) follow-ups. PPD reduction was also significantly greater in test group at 4 week period (-1.98 ± 0.55 vs -1.58 ± 0.56, test vs control, *P*=0.0224).

**Conclusions:**

Er,Cr:YSGG-assisted periodontal therapy is beneficial in MGI and PPD reductions during early healing period.

**Supplementary Information:**

The online version contains supplementary material available at 10.1186/s12903-024-04270-1.

## Introduction

The efficacy of laser application in periodontal treatment is still debatable. Various laser instruments are available in the market, and each instrument has its own working range of wavelength and energy level that originates from the type of medium utilized in the equipment. Therefore, various lasers that have been frequently used and studied in the dental field since the 1990s have different indications and characteristics, although they share general mechanisms. These include lasers such as carbon dioxide, neodymium-doped:yttrium, aluminum, and garnet (Nd:YAG), erbium-doped:yttrium, aluminum, and garnet (Er:YAG), erbium, chromium-doped:yttrium, scandium, gallium, and garnet (Er,Cr:YSGG), and diode lasers [1].

The Er,Cr:YSGG laser has a high absorption coefficient for water, especially OH^−^ ions, with a relatively high emission wavelength (2,780 nm). This causes the laser energy to be largely absorbed in the superficial layer of the tissue and the energy does not penetrate deeply into it. Energy absorbed water molecules evaporate easily by photo-thermal effects, increase intra-tissue pressure by vapors created within the tissue, provoking “micro-explosions” in the end that cause mechanical breakdown of the tissue. This series of processes is called tissue ablation [2, 3]. Unlike deep penetrating lasers, such as diodes and Nd:YAG lasers, erbium lasers do not exert secondary thermal effects during irradiation. Therefore, applying Er,Cr:YSGG laser in periodontal treatment, precisely at the stage of degranulation of periodontal pockets, enables the removal of inflamed tissue without causing unnecessary side effects, such as thermal damage and uncontrolled destruction of the underlying tissue. In addition, some plausible evidence suggests that bacteria colonizing within or beyond the pocket epithelium can be destroyed or inactivated by laser irradiation [4–8]. Therefore, laser-assisted periodontal treatment aids in the removal of calculus from deep pockets and enables elimination of infected pocket lining tissue as a direct step so that complete disinfection can be achieved far more reliably compared to that by conventional scaling and root planing (SRP) [1].

It is known that wound healing in laser-applied soft tissue surgery is delayed compared to that by scalpel incision because of the collateral thermal damage [9, 10]. However, Er,Cr:YSGG laser wounds are histologically reported to heal similar to scalpel wounds because of the minimal thermal effect and low tissue damage [11, 12].

Therefore, it is reasonable to anticipate that laser-assisted SRP will promote or accelerate the early healing phase and decrease inflammation recurrence. However, although there are numerous reports on long-term results, studies dealing with early healing phase results are scarce. It is meaningful to investigate early healing in periodontal treatment because it is associated with instantaneous relief of postoperative patient discomfort and decision making for subsequent surgical intervention. Therefore, this study aimed to demonstrate the efficacy of laser-applied periodontal treatment in promoting healing during the early phase. Cytokine analysis was performed in particular with the intention to examine the healing process more concretely [13].

## Materials and methods

### Study population

This study was designed as a split-mouth, single-blinded, randomized controlled clinical trial. Among the patients who visited the Department of Periodontology, Korea University Anam Hospital from May 2020 to April 2021, 13 patients aged 36–67 (mean age 52.2) years were recruited.16 quadrants per group were assigned through randomization. Quadrants assigned to test group were treated according to the laser-assisted periodontal treatment protocol, while control group was treated by conventional treatment modality. This study was conducted following ethical principles in accordance with the Declaration of Helsinki and approved by the Institutional Review Board of the Korea University Anam Hospital (IRB No. 2019AN0551). The study was registered at ClinicalTrials.gov (NCT05588544) on 20/10/2022. Each patient signed a written consent form before enrollment.

The inclusion criteria were as follows: (a) diagnosis of stage III or IV periodontitis based on the 2017 periodontitis classification [14]; (b) minimum of two teeth with probing depth (PD) > 5 mm in at least two quadrants of the whole dentition; (c) each quadrant with a minimum of four teeth; and (d) systemically healthy patients.

Patients were excluded if they had the following conditions: (a) history of periodontal treatment in the previous six months; (b) antibiotic medication in recent three months; (c) under steroid therapy or taking any anti-inflammatory drugs in recent three months; (d) history of any systemic disease that may influence the periodontal condition and treatment outcome, including diabetes mellitus, cancer, metabolic diseases, cardiovascular disease, and rheumatoid arthritis; (e) pregnancy or breastfeeding; and (f) smoking habits.

### Outcomes

The primary outcome of the study was modified gingival index (MGI) difference at 1, 2, 4, and 8w post-operatively. Secondary outcomes included differences in periodontal probing depth (PPD), clinical attachment level (CAL), and bleeding on probing (BOP) at 4 and 8w follow-up, and gingival crevicular fluid (GCF) cytokine (IL-1β, TGF-β, IL-8) level changes in each post-operative visit.

### Sample size calculation

The sample size calculation was based on the split-mouth study design [15]. The sample size formula used was as follows:

$${n}_{s}=\frac{2{\sigma }^{2}(1-\rho ){({z}_{1-\frac{\alpha }{2}}+{z}_{1-\beta })}^{2}}{k{\delta }^{2}}$$

where ${n}_{s}$ represents the number of patients, $\delta$ is the treatment mean difference (the primary outcome, MGI), ${\sigma }^{2}$ is the variance of $\delta$, $\rho$ is the correlation coefficient between outcomes in a patient, $k$ is the number of sites per segment, $\alpha$ is the two-sided level of significance, and $\beta$ is the type II error rate.

Using the two-sided significance level of 5%, assuming that the variance of clinical outcomes is 0.76 and a correlation coefficient between clinical outcomes in a patient is 0.3, the number of patients from a minimum of 5 (when four teeth are treated in four quadrants) to a maximum of 18 (when two teeth are treated in two quadrants) is needed to ensure a minimum of 80% power for detecting a treatment effect difference of 0.43 between groups when a drop-out rate of 10% is considered. The values of the minimum clinically important difference, variance, and correlation coefficient were estimated using the results of Cha et al. (2019) [16].

### Randomization and allocation concealments

Following the determination of patient quadrants, code-assigned sealed envelopes were opened. Each envelope contained a sequence of group assignments (control or test) previously generated with computer-based randomization. The PROC PLAN procedure in SAS (Ver. 9.4; SAS Institute, Cary, NC, USA) was used for randomization. Control quadrants were assigned to receive conventional SRP procedures, whereas test quadrants received laser-assisted SRP treatment. The allocation envelopes were restored in a locked cabinet and remained under the supervision of a separate investigator throughout the study. Patients were not informed which side was the test side.

### Test protocol

Periodontal treatment was performed by a single periodontist (J.S.P). SRP was performed using Gracey curettes and an ultrasonic scaler. Supra and subgingival calculi were meticulously removed from the root surfaces. For the test quadrants, an Er,Cr:YSGG laser (Waterlase Express™, Biolase, Inc., Foothill Ranch, CA) was applied during the SRP procedure. The details of each step are as follows:

1. De-epithelization of the internal pocket epithelium and retraction: Radial firing perio tip (RFPT) 5 (Biolase, Inc., Foothill Ranch, CA) (1.5 W, 40% air/50% water, 30 Hz, 50 mJ/pulse, energy density of 6 J/cm.^2^)
2. SRP with Gracey curettes and ultrasonic scaler
3. Sulcular debridement and degranulation: RFPT 5 (Biolase, Inc., Foothill Ranch, CA) (1.5 W, 40% air/50% water, 30 Hz, 50 mJ/pulse, energy density of 6 J/cm.^2^)
4. Outer pocket de-epithelialization: MZ 6 (1.5 W, 40% air/50% water, 30 Hz, 50 mJ/pulse, energy density of 6 J/cm.^2^)
5. Pressure with wet gauze for 1–2 min.

Patients were instructed to refrain from tooth brushing for 3 days after treatment. Interdental brushing was prohibited during 1 week after treatment. A chlorhexidine rinse was prescribed twice daily for 7 days. Patients were instructed to take painkillers (500 mg acetaminophen) if the pain was irresistible.

### Study procedures and outcome measures

At the first visit, patient screening was performed by means of a clinical examination. Prior to the consent for the study, patients were provided with detailed information regarding the study, including the study plan, intervention method, material characteristics, possible side effects, and patients’ right to refuse or discontinue study participation. Oral hygiene instructions were provided to the enrolled patients using disclosing solution and plaque control devices, including manual toothbrushes and interdental brushes.

On the day of treatment, baseline measurements were taken, and GCF samples were collected prior to treatment. Teeth having at least one site with PPD > 5 mm were subject for analysis. Clinical examination included PPD, gingival recession (REC), MGI [17], BOP, plaque index (PI), and mobility (MOB). The PPD and REC were measured (mm) at six sites per tooth. CAL referred to the distance between the cementoenamel junction and the base of the pocket. The MGI and PI were measured at four and two sites, respectively. GCF sampling and MGI, PI, and MOB measurements were performed at 1, 2, 4, and 8w post-operative visits. PPD, CAL, and BOP were measured at 4 and 8w. At each follow-up visit, supragingival plaque control was performed using ultrasonic scaler after clinical measurement and GCF sampling were done. Data measurements were conducted by a blinded researcher who was not engaged in the treatment procedure. UNC15 probe was used in measurements. The calibration process was conducted prior to study execution until the repeated measurements has substantial correlation calculated by Cohen’s Kappa (k ≥ 0.6). ln addition to the Kappa agreement, the measurements were required to show a 90% agreement for ± 1 mm.

### GCF sampling

GCF samples were collected from the deepest periodontal pockets of each subject tooth. Two paper strips (PerioPaper, Oraflow, Plainview, NY, USA) were used sequentially to collect GCF from one periodontal site. The sampling areas were air-dried and isolated with cotton rolls to prevent contamination by saliva. The strips were then inserted into the gingival crevice until mild resistance was felt and left in place for 30 s. Strips contaminated with blood were discarded. Two paper strips from the same pocket were placed into a sterile polypropylene 1.5 mL microtube filled with 300 µL phosphate-buffered saline. After being eluted at 4℃ overnight, samples were centrifuged at 400 g for 4 min, supernatants were separated and transferred to a new microtube to be stored at -80℃ until assayed.

### GCF analysis

Multiplex cytokine analysis was performed. The Luminex-100 system is a flow-cytometry-based multiplex protein analysis system widely used in biomarker research. The system uses microparticles precoated with analyte-specific antibodies. These magnetic microparticles are embedded with fluorophores at set ratios to classify discrete beads. Following covalent coupling of the beads to the analyte of interest, a second detection antibody is reacted to quantify the amount of analyte bound to the beads. This secondary antibody is directly conjugated to biotin and then reacted with streptavidin–phycoerythrin (streptavidin-PE). Light emitting diodes (LEDs) from the analyzer excite the dyes inside each microparticle to identify the microparticle region and excite the PE to measure the amount of analyte bound to the microparticle [18].

### Statistical analyses

All numeric variables are summarized as means ± standard deviations. Comparisons of measured variables between the test and control groups were made using Generalized estimating equation model considering clustered data. Changes from baseline to 1, 2, 4, and 8 weeks of follow-up were examined by repeated measures analysis of variance (ANOVA) considering clustered data. All statistical analyses were performed using SAS software (version 9.4; SAS Institute, Cary, NC, USA). *P*-value < 0.05 was considered statistically significant.

## Results

**Fig. 1:**
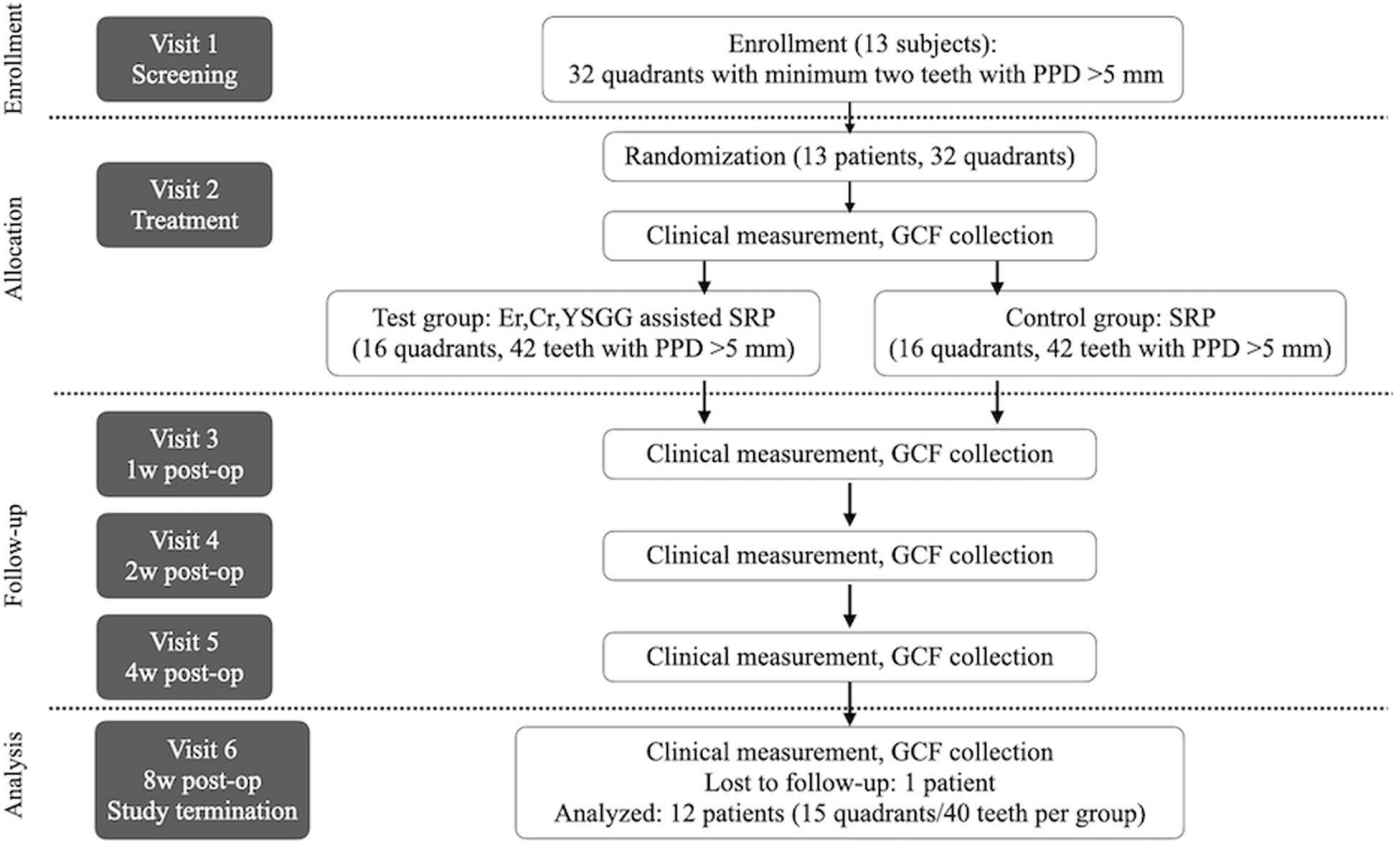
Flow chart of the study

Twelve patients (mean age 52.2 years), 15 quadrants 68 (control)/67 (test) teeth per group were included in the treatment procedure. Among them, 40 teeth per group (control/test) with at least one site having PPD > 5 mm were subject to the statistical analysis (Fig. 1). While 13 patients were originally enrolled, one patient dropped out at the 3rd visit.

There was no statistical difference in the baseline clinical values between the control and test groups, except for BOP, which showed slightly higher values in the test group (Table 1). No adverse outcomes were reported in the study, and no patient reported taking medication due to pain during the entire study period.

**Table 1:** Baseline clinical measurements

Variable	n	Mean (SD)	P-value*
**PPD**			0.9693
Test	40	5.00 (0.97)	
Control	40	5.00 (0.99)	
**CAL**			0.2633
Test	40	5.49 (1.10)	
Control	40	5.80 (1.43)	
**MGI**			0.468
Test	40	2.81 (0.43)	
Control	40	2.74 (0.50)	
**BOP**			0.0213
Test	40	0.90 (0.19)	
Control	40	0.77 (0.32)	
**PI**			0.7978
Test	40	1.40 (0.79)	
Control	40	1.45 (0.95)	

*PPD* Probing pocket depth, *CAL* Clinical attachment level, *BOP* Bleeding on probing, *MGI* Modified gingival index, *PI* Plaque index, *SD* Standard deviation

^*^P-value by generalized estimating equation model considering clustered data

### PPD, CAL, and MGI changes on the whole subject teeth

In the initial statistical analysis on the whole subject teeth, clinical variables including PPD, CAL, and MGI showed significant decrease throughout 8w of follow-up period compared to the baseline regardless of the treatment modality (Table 2). At 1w, changes of MGI values were significantly greater in test group (-1.07 ± 0.45 vs -0.80 ± 0.66, test vs control, *P* = 0.0153). At 4w, reduction in both mean PPD (*P* = 0.0253) and MGI (*P* = 0.0318) were significantly greater in test group. While CAL showed lower mean value at 4w in test group groups (4.06 ± 1.02 mm vs 4.59 ± 1.25 mm, test vs control, *P* = 0.0339), amount of CAL gain compared to the baseline was comparable to the control (1.43 ± 0.94 vs 1.21 ± 0.79, test vs control, *P* = 0.2524)). At 8w follow-up, MGI reduction compared to the baseline was significantly greater in test group compared to the control (-1.69 ± 0.65 vs -1.25 ± 0.77, test vs control, *P* = 0.0047). Difference in PPD reduction at 8w showed marginal significance (-1.74 ± 0.68 vs -1.46 ± 0.64, test vs control, *P* = 0.0526) (Table 2).

**Table 2:** Results on the whole subject teeth. Mean values and changes for PPD, CAL, and MGI

Variable	N	Baseline	1w		2w		4w		8w	
			**Mean (SD)**	$\Delta \boldsymbol{ }(\mathbf{S}\mathbf{D})$	**Mean (SD)**	$\Delta \boldsymbol{ }(\mathbf{S}\mathbf{D})$	**Mean (SD)**	$\Delta \boldsymbol{ }(\mathbf{S}\mathbf{D})$	**Mean (SD)**	$\Delta \boldsymbol{ }(\mathbf{S}\mathbf{D})$
**PPD**										
Test	40	5.00 (0.97)					3.33 (0.73)^a^	-1.67 (0.59)^b^	3.25 (0.69)^a^	-1.74 (0.68)^c^
Control	40	5.00 (0.99)					3.64 (0.91)^a^	-1.37 (0.63)	3.54 (0.76)^a^	-1.46 (0.63)
**CAL**										
Test	40	5.49 (1.10)					4.06 (1.02)^ab^	-1.43 (0.94)	4.07 (1.00)^a^	-1.42 (1.01)
Control	40	5.80 (1.43)					4.59 (1.25)^a^	-1.21 (0.79)	4.48 (1.31)^a^	-1.33 (0.84)
**MGI**										
Test	40	2.81 (0.43)	1.70 (0.53)^a^	-1.07 (0.45)^b^	1.41 (0.82)^a^	-1.42 (0.67)	1.31 (0.75)^a^	-1.51 (0.67)^b^	1.12 (0.71)^ab^	-1.69 (0.65)^b^
Control	40	2.74 (0.50)	1.93 (0.53)^a^	-0.80 (0.66)	1.52 (0.83)^a^	-1.22 (0.85)	1.55 (0.79)^a^	-1.19 (0.67)	1.79 (0.77)^a^	-1.25 (0.77)

*PPD* Probing pocket depth, *CAL* Clinical attachment level, *MGI* Modified gingival index, *SD* Standard deviation

^a^Statistically significant difference in comparison to the baseline value (*P* < 0.05, analyzed using repeated measure ANOVA considering clustered data)

^b^Statistically significant difference in comparison to the control group in the same period (*P* < 0.05, analyzed using Generalized estimating equation model considering clustered data)

^c^Marginally significant difference in comparison to the control group in the same period (*P* = 0.0526, analyzed using Generalized estimating equation model considering clustered data)

### PPD, CAL, and MGI changes on the teeth with mean PPD ≥ 5 mm

When teeth with mean PPD ≥ 5 mm were sorted, CAL differences between the control and test groups were more apparent. At 4w, CAL values were 4.27 ± 1.05 mm and 5.25 ± 1.27 mm (test and control, respectively, *P* = 0.006). CAL gain compared to the baseline was greater in test group at 4w and the difference showed marginal significance (-1.79 ± 0.94 vs -1.34 ± 0.67, test vs control, *P* = 0.0717). CAL values at 8w were 4.21 ± 1.00 mm and 5.12 ± 1.25 mm (test and control, respectively, *P* = 0.0094), but the amount of CAL gain showed no significant difference (Table 4). MGI decrease was significantly greater in test group at 1 (*P* = 0.003) and 8w (*P* = 0.0102) follow-ups. PPD reduction was also significantly greater in test group at 4w period (-1.98 ± 0.55 vs -1.58 ± 0.56, test vs control, *P* = 0.0224) (Table 4).

Results on BOP and PI changes are shown in supplement table (Table S1). BOP significantly decreased from the baseline value throughout 8w period, but intergroup difference was non-significant. In terms of cytokine analysis, concentration of IL-1β, TGF-β1, and IL-8 showed tendency to decrease in general, however, statistical significances were not shown due to large standard deviations. Intergroup differences were not apparent either (Tables 3 and 5).

**Table 3:** Results on the whole subject teeth. Cytokine analysis

Variable	n	Baseline	1w	2w	4w	8w
**IL-1β**						
Test	40	690.78 (461.27)	609.57 (438.24)	503.54 (451.10)	484.08 (397.28)^a^	538.00 (536.74)
Control	40	742.35 (467.04)	534.22 (503.34)	531.27 (378.40)^a^	477.25 (349.18)^a^	411.99 (229.92)^a^
**TGF-β1**						
Test	40	71.91 (86.03)	53.54 (49.37)	44.38 (90.06)	18.34 (33.40)^a^	24.69 (29.80)^a^
Control	40	128.04 (160.37)	46.79 (86.40)^a^	21.12 (23.06)^a^	23.45 (4.21)^a^	25.24 (43.21)^a^
**IL-8**						
Test	40	600.59 (316.99)	873.82 (601.03)^a^	470.45 (308.76)	559.68 (348.45)	453.36 (286.04)^a^
Control	40	657.97 (704.94)	706.53 (406.57)	464.53 (267.44)	556.68 (386.63)	399.05 (247.48)^a^

^a^Statistically significant difference in comparison to the baseline value (analyzed using repeated measure ANOVA considering clustered data)

## Discussion

Overall, Er,Cr:YSGG laser application in periodontal treatment was beneficial for decreasing PPD, CAL, and MGI values in 8w healing period. MGI reductions were significantly greater in test group at 1, 4, and 8w compared to control (*P* < 0.05, Table 2). PPD reduction was also greater in test group at 4 (-1.67 ± 0.59 vs -1.37 ± 0.63, test vs control, *P* < 0.05) and 8w (-1.74 ± 0.68 vs -1.46 ± 0.64, test vs control, *P* = 0.0526). Differences in CAL values were more evident when teeth with advanced periodontal damage were sorted (mean PPD ≥ 5 mm). CAL gain at 4w was greater in test group and the difference showed marginal significance (-1.79 ± 0.94 vs -1.34 ± 0.67, test vs control, *P* = 0.0717). Amount of PPD reductions at 4w were -1.98 ± 0.55 (test) and -1.58 ± 0.56 (control) showing significant difference between two groups (*P* < 0.05). MGI decrease was significantly greater in test group at 1 and 8w (*P* < 0.05) (Table 4). GCF concentration of IL-1β cytokine showed continuous decrease in the test group and dropped remarkably between 4 and 8w while that in the control group maintained after 1w postoperative (Table 5). These results suggest that the Er,Cr:YSGG laser is beneficial for reducing inflammation in early healing period when applied in periodontal treatment.

**Table 4:** Analysis on the teeth with mean PPD ≥ 5 mm. Mean values and changes for PPD, CAL, and MGI

Variable	n	Baseline	1w		2w		4w		8w	
			**Mean (SD)**	$\Delta \boldsymbol{ }(\mathbf{S}\mathbf{D})$	**Mean (SD)**	$\Delta \boldsymbol{ }(\mathbf{S}\mathbf{D})$	**Mean (SD)**	$\Delta \boldsymbol{ }(\mathbf{S}\mathbf{D})$	**Mean (SD)**	$\Delta \boldsymbol{ }(\mathbf{S}\mathbf{D})$
**PPD**										
Test	20	5.71 (0.82)					3.73 (0.75)^a^	-1.98 (0.55)^b^	3.63 (0.71)^a^	-2.08 (0.64)
Control	20	5.75 (0.73)					4.17 (0.91)^a^	-1.58 (0.56)	4.00 (0.75)^a^	-1.75 (0.62)
**CAL**										
Test	20	6.06 (1.05)					4.27 (1.05)^ab^	-1.79 (0.94)^c^	4.21 (1.00)^ab^	-1.85 (0.96)
Control	20	6.59 (1.16)					5.25 (1.27)^a^	-1.34 (0.67)	5.12 (1.25)^a^	-1.47 (0.82)
**MGI**										
Test	20	2.88 (0.46)	2.14 (0.46)^ab^	-1.19 (0.40)^b^	1.74 (0.84)^a^	-1.16 (0.71)	1.85 (0.74)^a^	-1.21 (0.53)	1.90 (0.61)^a^	-1.46 (0.62)^b^
Control	20	2.95 (0.46)	1.79 (0.44)^a^	-0.74 (0.52)	1.79 (0.87)^a^	-1.14 (0.79)	1.74 (0.71)^a^	-1.03 (0.53)	1.49 (0.74)^a^	-0.98 (0.61)

*PPD* Probing pocket depth, *CAL* Clinical attachment level, *MGI* Modified gingival index, *SD* Standard deviation

^a^Statistically significant difference in comparison to the baseline value (*P* < 0.05, analyzed using repeated measure ANOVA considering clustered data)

^b^Statistically significant difference in comparison to the control group in the same period (*P* < 0.05, analyzed using Generalized estimating equation model considering clustered data)

^c^Marginally significant difference in comparison to the control group in the same period (*P* = 0.0717, analyzed using Generalized estimating equation model considering clustered data)

**Table 5:** Analysis on the teeth with mean PPD ≥ 5 mm. Cytokine analysis

Variable	n	Baseline	1w	2w	4w	8w
**IL-1β**						
Test	20	750.29 (435.67)	647.69 (596.35)	588.32 (352.11)	543.62 (380.39)	393.95 (255.43) ^a^
Control	20	858.80 (496.37)	604.07 (351.69)	562.27 (504.51)	525.34 (376.55)^a^	607.68 (499.34)
**TGF-β1**						
Test	20	149.71 (151.18)	61.03 (114.10)^a^	22.35 (20.87)^a^	21.83 (21.68)^a^	28.77 (41.89)^a^
Control	20	108.89 (107.33)	77.41 (55.14)^a^	53.42 (105.39)^a^	22.64 (42.78)^a^	32.33 (34.71)^a^
**IL-8**						
Test	20	674.89 (323.61)	963.50 (690.61)	499.39 (379.52)	597.20 (330.49)	526.05 (308.49)
Control	20	669.96 (609.01)	777.81 (406.95)	455.67 (237.24)	595.54 (483.66)	347.55 (190.36)^a^

^a^Statistically significant difference in comparison to the baseline value (*P* < 0.05, analyzed using repeated measure ANOVA considering clustered data)

When comparing the results with those of previous reports, it is important to consider the severity of disease at the periodontal sites, as well as details of the treatment protocol, including the tip being used. Laser-assisted periodontal treatment procedures include de-epithelization and retraction, sulcular debridement and degranulation, and outer pocket de-epithelization within the protocol. De-epithelization and retraction performed prior to calculus removal expose the gingival sulcus, allowing visual access and a better approach to the deeper parts of the periodontal pocket. Sulcus debridement using an RFPT enables effective removal of inflammatory tissue from the inner lining of the pocket because of the radial emission of laser energy. The RFPT5-14 fiber tip with a diameter of 580 μm and length of 14 mm as used in this study is particularly useful in multi-rooted teeth and periodontal sites where narrow and deep pockets are present because their size and material properties provide proper accessibility and flexibility to those regions.

However, there is a lack of published results on periodontal therapy using the Er,Cr:YSGG laser + RFPT protocol. Ustun et al. (2018) used RFPT5-14 and reported no significant difference in terms of clinical parameters and GCF changes between SRP alone and SRP + Er,Cr:YSGG laser [19]. Baseline PPDs in this study were considerably lower (3.880 ± 0.496 mm for control and 3.970 ± 0.722 mm for test group) compared to our study (5.00 ± 0.99 mm for control and 5.00 ± 0.97 mm for test group). In another study where RFPT5-14 was also used, there was significant difference in PPD measurement at 1-month follow-up (2.7 ± 0.4 mm and 2.3 ± 0.8 mm for control and test groups, respectively) during the total 6 m follow-up period [20]. Given that CAL at baseline was 2.9 ± 0.4 (control) and 2.0 ± 0.2 (test), the patients’ teeth in that study seemed to have had mild to moderate stage of periodontal disease. In a study by Ge et al. (2017), molars with furcation defects (class II or III) were treated with either Er,Cr:YSGG laser or hand instruments and reported significantly greater reductions in PPD and BOP in the laser-treated group at weeks 6 and 12 postoperatively [21]. Another study published by Clem et al. (2021) showed the non-inferiority of Er,Cr:YSGG laser therapy in terms of CAL and PD improvements at ≥ 6 mm depth intrabony defects when compared to surgical treatment using a minimally invasive surgical technique [4].

The first phase of conventional periodontal treatment, that is, SRP, involves the removal of bacterial deposits from the root surface facing the periodontal pocket. The transition from inflammatory to sound periodontal tissue occurs spontaneously following decontamination of the root surface. However, lesions with deep periodontal pockets often show unsatisfactory results with recurrence of inflammation, mostly due to incomplete removal of the subgingival calculus and residual pockets, which lead to a consequential decision for surgical treatment.

In pockets with PDs of > 6 mm, a higher percentage of calculus (up to 44%) remained after nonsurgical periodontal therapy compared that with shallower pockets having PDs of 4–5 mm (up to 29%) [22]. In a meta-analysis performed by Heitz-Mayfield et al. (2002), the open-flap debridement procedure was proven to outrank SRP alone in terms of PPD reduction (0.6 mm) and CAL gain (0.2 mm) in pockets with PPDs > 6 mm [23]. These reports indicate that better access to anatomical complexity alone can be beneficial for improving therapeutic outcomes in deep pockets. The concept of “critical probing depth” is also in line with these results. According to this well-established concept, 5.4 mm is a critical probing depth value above which access flap surgery will result in more CAL gain compared to that by SRP [24].

In our results, when teeth with an average PPD ≥ 5 mm were separately sorted, differences in CAL values between SRP alone and laser-assisted SRP were more apparent. Providedthat PPDs were measured from six sites per tooth, teeth sorted based on this criterion had at least one site with PPD > 6 mm. This indicates that teeth that may require subsequent surgical periodontal therapy due to severe attachment loss benefited remarkably from laser-assisted therapy. In terms of difference in CAL gain, laser + SRP was advantageous by approximately 0.4 mm compared to SRP alone. This number is not much different from the effectiveness of surgical therapy over SRP alone in sites with PPD > 6 mm as published in a previous meta-analysis, indicating that Er,Cr:YSGG combined with periodontal therapy exerts an influence corresponding to access flap surgery in advanced periodontal lesions [23]. However, our result should be interpreted with caution due to the marginal significance the intergroup difference had shown.

The purpose of cytokine analysis was to compare the activity of inflammation and wound healing objectively and numerically. However, it was found that the concentration of cytokines varies considerably among individuals as well as teeth within a patient, showing large standard deviations in cytokine concentrations. Previous studies have shown that although periodontal inflammation caused by microbial infection can raise the level of inflammatory cytokines in GCF, individual differences in the immunologic response, either hereditary or epigenetic, bring about significant variance in the concentration itself [25–28]. In this context, our results are consistent with those of previous studies. Despite the large variances, IL-1β data showed a clear tendency of decreased levels after treatment in both groups, demonstrating that SRP, either with or without adjunctive laser treatment, contributed to the resolution of inflammation. However, the decrease was more obvious in the test group with deeper pockets (teeth with mean PPD ≥ 5 mm) because the concentration continued to decrease throughout the study period in the test group, while it appeared to rebound at 8w in the control group (Table 5). This implies that laser application in periodontal treatment may assist in the removal of microbial endotoxins that would otherwise cause re-infection at the periodontal site, which bears the necessity for further surgical therapy.

Limitation of the study is the short study period. Although we intended to focus on the early healing, longer follow-up data should assist in the interpretation of the results we saw in this study and be more meaningful in determining the clinical efficacy of laser application in periodontal treatment. In addition, larger sample size may help in clarifying the differences seen in this study.

In conclusion, Er,Cr:YSGG-assisted periodontal therapy using RFPT is beneficial in MGI and PPD reductions when comparing to SRP alone. In teeth with an average PPD ≥ 5 mm, CAL gain may also be advantageous by applying Er,Cr:YSGG laser in combination with SRP. Further studies are needed to confirm the beneficial effect of laser-assisted periodontal therapy.

### Supplementary Information

**Supplementary material 1.**

## Data Availability

The datasets used and/or analysed during the current study are available from the corresponding author on reasonable request.
