# Supplementary material for: Effect of erbium, chromium-doped: yttrium, scandium, gallium, and garnet laser-assisted periodontal therapy using radial firing tip during early healing period: a randomized controlled split-mouth clinical trial
Source: BMC Oral Health. 2024 May 8;24:539. doi: 10.1186/s12903-024-04270-1 (PMC11080134; doi:10.1186/s12903-024-04270-1)
Supplement: Supplementary file 1 — Supplementary material 1. [file 12903_2024_4270_MOESM1_ESM.pdf]

Supplement

Table S1. Clinical measurements for BOP and PI

(a) All sites

Variable	N	Baseline	1w	2w	4w	8w
**BOP**						
Test	40	0.90 (0.19)			0.47 (0.33) *	0.40 (0.30) *
Control	40	0.77 (0.32)			0.53 (0.27) *	0.48 (0.27) *
**PI**						
Test	40	1.4 (0.79)	0.5 (0.63) *	0.74 (0.71) *#	0.94 (0.81) *	0.88 (0.90) *
Control	40	1.45 (0.95)	0.66 (0.67) *	0.41 (0.55) *	0.99 (0.80) *	1.08 (0.97) *

*: Statistically significant difference in comparison to the baseline value (P <0.05, analyzed using repeated measure ANOVA considering clustered data)

#: Statistically significant difference in comparison to the control group in the same period (P <0.05, analyzed using Generalized estimating equation model considering clustered data)

PI, plaque index; BOP, bleeding on probing

(b) Teeth with mean PPD ≥5 mm

Variable	n	Baseline	1w	2w	4w	8w
**BOP**						
Test	20	0.90 (0.21)			0.61 (0.32) *	0.55 (0.31) *
Control	20	0.84 (0.32)			0.60 (0.24) *	0.60 (0.23) *
**PI**						
Test	20	1.55 (1.18)	0.65 (0.73) *	0.28 (0.44) *#	1.13 (0.93)	1.28 (0.92) *
Control	20	1.48 (0.82)	0.55 (0.67) *	0.65 (0.69) *	1.10 (0.84)	1.00 (0.97)

*: Statistically significant difference in comparison to the baseline value (P <0.05, analyzed using repeated measure ANOVA considering clustered data)

#: Statistically significant difference in comparison to the control group in the same period (P <0.05, analyzed using Generalized estimating equation model considering clustered data)

PI, plaque index; BOP, bleeding on probing
